# Ants regulate colony spatial organization using multiple chemical road-signs

**DOI:** 10.1038/ncomms15414

**Published:** 2017-06-01

**Authors:** Yael Heyman, Noam Shental, Alexander Brandis, Abraham Hefetz, Ofer Feinerman

**Affiliations:** 1 Department of Physics of Complex Systems, Weizmann Institute of Science, Rehovot 7610001, Israel; 2 Department of Computer Science, The Open University of Israel, Raanana 4353701, Israel; 3 Faculty of Biochemistry, Weizmann Institute of Science, Rehovot 7610001, Israel; 4 Department of Zoology, Tel Aviv University, Tel-Aviv 69978, Israel

## Abstract

Communication provides the basis for social life. In ant colonies, the prevalence of local, often chemically mediated, interactions introduces strong links between communication networks and the spatial distribution of ants. It is, however, unknown how ants identify and maintain nest chambers with distinct functions. Here, we combine individual tracking, chemical analysis and machine learning to decipher the chemical signatures present on multiple nest surfaces. We present evidence for several distinct chemical ‘road-signs' that guide the ants' movements within the dark nest. These chemical signatures can be used to classify nest chambers with different functional roles. Using behavioural manipulations, we demonstrate that at least three of these chemical signatures are functionally meaningful and allow ants from different task groups to identify their specific nest destinations, thus facilitating colony coordination and stabilization. The use of multiple chemicals that assist spatiotemporal guidance, segregation and pattern formation is abundant in multi-cellular organisms. Here, we provide a rare example for the use of these principles in the ant colony.

## Abstract (web summary)

While the organization of ants within their nest is key for colony function, it remains unknown how ants navigate this dark subterranean environment. Here, Heyman *et al*. use a series of behavioral tests, chemical analyses, and machine learning to identify chemical landmarks that ants use to distinguish between nest areas.

Social insects live in self-organized groups that lack centralized control^1^,^2^,^3^. This lack of global directives requires each individual to act upon local information, either personal or social, which is accessible at its immediate environment. Ants that are in spatial proximity to a location where work is required indeed tend to contribute to the desired task^4^ and the sum of many such individual decisions impact colony scale division of labour^5^. The location of an ant further determines whom she interacts with^6^,^7^,^8^,^9^ therefore influencing her social information. This is of utter importance since ants heavily rely on interactions in their subsequent decisions towards collective colony goals^1^,^10^. The spatio-temporal distribution of ants within the nest is, therefore, a key component of the collective behaviour of the colony.

Evidence shows that the distribution of ants within their nest is non-random. For example, in natural nests of *Pogonomyrmex badius* the majority of nurses are found deep within the nest, while foragers tend to be close to the surface^11^. Order is also maintained in artificial, two-dimensional lab nests. There, different workers have been observed to spend a large part of the time in specific nest areas, appropriately termed as ‘spatial fidelity zones'^4^,^7^. However, little is known of the mechanisms that allow ants to recognize their preferred location inside the nest.

Outside the nest, ants are known to find their way using a variety of mechanisms including path integration, landmark recognition, magnetic sensing, light polarization, chemical cues and pheromone trails^12^,^13^,^14^,^15^,^16^,^17^,^18^. Using cues that are suitable outside the nest for subterranean orientation is not straightforward: the nest's dark environment prevents the use of vision and its complex architecture renders the use of spatial memory unlikely. Moreover, CO_2_ soil gradients, hypothesized to contain depth information, were not found to be used as navigational cues^19^. Finally, while gravitation does supply vertical directionality, distinguishing between locations that lie on the same horizontal plane must be achieved through other mechanisms.

A key mechanism that facilitates social group organization is stigmergy—indirect communication between individuals mediated through the environment^20^. Stigmergic processes enable long-range interactions and underlie collective memory. In ant colonies, stigemergic interactions often involve chemical marking of the environment^18^,^21^,^22^. A well-known example is that of the pheromone trail which labels the path between a food source and the nest^18^. The pheromonal signatures on the ant trail are complex and may code the current state of the trail (such as exploration versus exploitation)^23^, the distance from the nest^24^,^25^ and even provide negative ‘no-entry' signals^26^. Furthermore, it has been shown that ants can identify the scent of their nest soil^27^. Indeed, colony specific cuticular hydrocarbons have been identified within the nest^25^,^28^. This richness of chemical cues suggests their stigmergic function as a possible means for orientation and positioning within the nest.

Here, we show that the surfaces of laboratory nests of the carpenter ant, *Camponotus fellah,* are labelled with chemical ‘road-signs', which we define as spatially localized information-bearing chemical features that directly affect the ants' movements inside the dark nest. We start by establishing that the spatial organization of the colony emerges from the spatial fidelity of different ants to different areas in the nest. Then, we show that the ants' organization within their nest can be manipulated by shuffling the locations of the chambers' floors. These results suggest a relation between ant orientation and certain attributes that are imprinted onto the nest surfaces. Next, we show that these attributes are chemical in nature and that their hydrocarbon components serve an important role. Moreover, we find that the hydrocarbon extracts collected from chambers of different functionality have distinct chemical signatures and provide evidence for several distinguishable signatures. Using active manipulation experiments, we show that ants of different task groups indeed exhibit discriminative reactions to three of these chemical signatures. Finally, we track the source of these chemicals to show that road-signs are not the simple by-product of ants that passively shed their cuticular hydrocarbons onto the surfaces of the chamber at which they reside.

## Results

### Ants have preferred locations within the nest

Outside the nest, most individual ants follow pheromone trails by aligning with them and traveling along their path. Hence, in this scenario, the spatial distribution of pheromones is readily ‘visible' as the distribution of all nearby ants. However, this is not the case within the nest where ants engaged in different tasks may react differently to the same orientation cues. Here, the spatial distribution of non-identified ants holds little information regarding the chemical substrate. To overcome this difficulty, and study the ants' spatial organization within the nest, we tracked colonies in which all ants were individually tagged^7^,^29^ (Bugtag, Robiotec).

**Figure 1:**
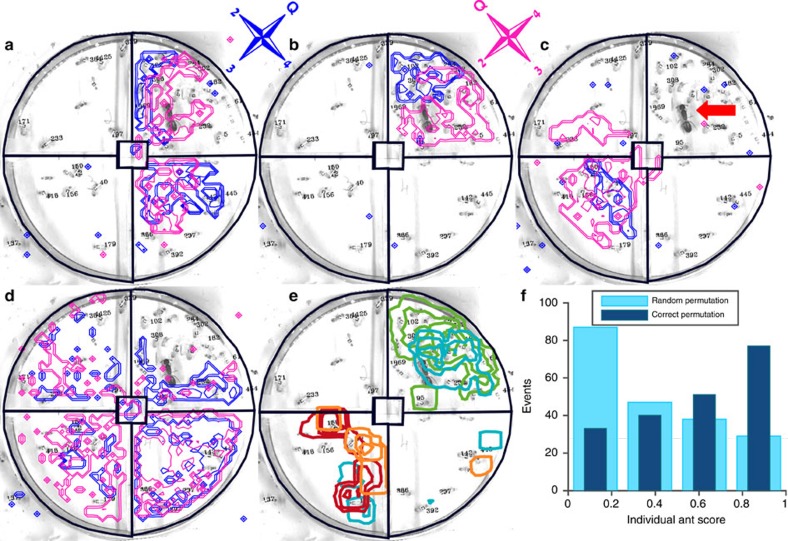
Ants identify and return to their preferred location in the nest. (**a**–**d**) Blue lines depict contour plots of the 2D location histograms of four sample ants overlaid on an image of the symmetrical four chamber nest which they occupied. The square frame in the middle of each structure is an entrance through its infrared filter top. The blue compass indicates the position of the queen's chamber (queen is marked by red arrow which also serves as a 2 cm scale bar in **c**). Magenta contours indicate the locations of the same four ants in a new identical structure in which floor locations were rotated as depicted by the magenta compass. The orientation of the magenta contours is corrected to cancel the rotation and emphasize the overlap with ant locations before the manipulation. (**e**) Contour plots of the 2D histogram of the cumulative locations of ants who spent over 70% of their time in chamber 1 (15 ants) and chamber 3 (5 ants) before (green and red, correspondingly) and after (blue and orange rotated histograms) the manipulation. (**f**) Dark blue—A histogram of individual ant (*N*=228 ants in 6 experiments) scores (quantifying the degree to which ants return to occupy the same floor segments as they had before the manipulation) calculated for the correct association between floor segments and their locations in the new nest (for example, a simple rotation by 90° in the experiment depicted in **a**–**e**). Light blue—individual ant score distribution calculated for random associations. A score of one indicates ants that spent an equal fraction of time on the same floors before and after the manipulation. A score of zero implies that an ant switched its floor preferences between the two stages of the experiment.

We started by verifying that, similar to previous findings^4^,^7^, *C. fellah* exhibit spatial fidelity to different areas of their nest. To do this, we introduced ant colonies into a 2D nest consisting of four identical chambers with paper floorings (the nest's structure is depicted in Fig. 1a–e and a scheme of the nest structure appears in Supplementary Fig. 1). We then continuously recorded the locations of all ants during a five day period. We find that ants that spent at least 75% of their time within the nest (*N*=228 ants in six experiments using 3 colonies) spent most of their time (an average of 71%) in a single chamber. Chamber preferences differ for different ants (for examples, see Fig. 1a–d). Trivially, completely immobile ants would have exhibited high fidelity to a single chamber, but this is not the case here. During the course of these experiments the ants often (a median of 7 times) moved outside their preferred chamber only to return to it later. Similarly, ants that left the nest (*N*=40 ants in 6 experiments) returned to the same chamber they exited with a probability of 0.4, significantly above the 0.25 random case (*P*<0.012, by the weight of the tail of the corresponding Binomial distribution). These observations indicate that nest inhabitants spatially segregate within the nest and have a preferred location which they recognize and are able to orient and return to.

### Nest surfaces affect ant spatial organization

Next, we performed behavioural manipulations designed to discern whether, to maintain spatial fidelity, ants use spatial memory or landmarks imprinted onto the surfaces of the nest

Ants were allowed to freely roam within the nest during the five-day ‘priming stage' described above. Following this priming period, we removed the flooring segments from the different nest chambers and, after shuffling their positions, inserted them into a new identical nest (*N*=6 experiments using 3 colonies with each colony used twice). Floor segment shuffling was done either randomly (*N*=2 out of 6) or by ∼90 degrees rotation relative to the segments previous orientation; As these gave similar results (see Supplementary Fig. 2) they were analysed together. The colony was then allowed to emigrate into the new nest (Supplementary Movie 1) while taking precautions to ensure that if the ants are able to return to their original locations within the nest then this could only be attributed to properties of the paper floors that had been acquired during the priming period due to the ants' presence (see Methods).

We compared the ants' locations within the nest before and after the manipulation. We find that most ants returned, not to the location of their previous chamber but, rather, to the same floor segment they resided on prior to the manipulation (Fig. 1a–e). Importantly, although the majority of the population resides in the queen's chamber and ants exhibit higher fidelity to this chamber (Supplementary Note 1), it appears that the ants' response cannot be attributed to a single stimulus (associated with the queen's chamber's floor). Rather, ants that occupied chambers other than the queen's before the manipulation also showed high fidelity to their original floor segment. This finding supports the existence of at least two signals (Fig. 1e, red and orange contours).

To demonstrate the significance of this effect, we defined a score that quantifies the degree to which ants return to occupy the same floor segments as they had before the manipulation (see the Methods section for details of the analysis). To quantify how the experimentally measured score deviated from chance, we compared it with computer generated scores of permuted ‘experiments' in which floor segments are incorrectly assigned to their locations within the new nest (see Methods section ‘Permutation test' and Supplementary Methods). We find that the score of the actual experimental data is large compared to the permuted data (Fig. 1f). This statistically supports the fact that the ants rely on floor characteristics when they returned to the nest (*P*<4e−6).

Furthermore, a similar analysis indicates that floor markings must contain, not just one but at least two different stimuli conveying positional information (*P*<2e−2, Supplementary Methods). Indeed, the presence of these two distinct stimuli is clearly visible in the ant distributions before and after the manipulation as presented in Fig. 1e. Following a large perturbation, these stimuli allow the colony to recover and return to its original spatial configuration and can therefore be understood to stabilize the colony's distribution. Given the fact that ant associated chemicals were previously identified within the nest^25^,^27^,^28^ it is natural to assume that these stimuli are of a chemical nature. In the next section, we test this assumption.

### Hydrocarbons as subterranean road-signs

Hydrocarbons have been shown to play a key role in ant communication^30^. To test the role of these substances as chemical stimuli within the nest we removed them (along with all other hexane soluble compounds) from nest floors by rinsing with hexane and observed the effect on ant organization.

Colonies (*N*=9) were placed in a symmetrical four chamber nest (Supplementary Fig. 1) with paper floorings in which three chambers were blocked. After 5 days, the floor of each of the four chambers was subjected to one of two treatments: (1) drying under a nitrogen stream (*N*=4); (2) rinsing by sonication in 200 ml of hexane followed by drying under a nitrogen stream (*N*=5). The unrinsed and rinsed floors were each transferred to new nest structures in which all chambers were accessible to the ants. The ants were then introduced to each of the new nests and the number of ants in each chamber was evaluated after 24 h. Each colony was subjected to both treatments at a random order with, at least, one week between treatments.

In all experiments with unrinsed floors, the chamber that contained the largest fraction of ants (40–80% of the colony, see Supplementary Note 2) was the chamber that was originally accessible and presumably marked. This implies that the first treatment did not remove the orientation stimulus (*P*<4e−3, by the weight of the tail of the corresponding Binomial distribution). Identical results were obtained for a similar experiment using unrinsed silica floors (Supplementary Note 3). On the other hand, in the rinsed conditions, the previously accessible chamber contained the largest fraction of ants in only one out of five cases, which can be expected in case the room choice is random (*P*=0.3955, by the weight of the tail of the corresponding Binomial distribution).

We further found that ants react to hexane extractions of their refuse area floor by recreating their new refuse pile on top of it (perfect match was observed in 25% of the experiments, see Supplementary Note 4). Taken together, the results presented thus far show that hexane soluble compounds act as chemical road-signs within the ants' nest.

### Classifying chamber function by its chemical signature

The evidence provided in the last two sections led us to expect chambers of similar functionality to have a similar hydrocarbon profile. To test this hypothesis, we extracted the hexane soluble signatures from different nest surfaces, measured their chemical composition and searched for correlations between the surfaces of chambers that share the same function.

**Figure 2:**
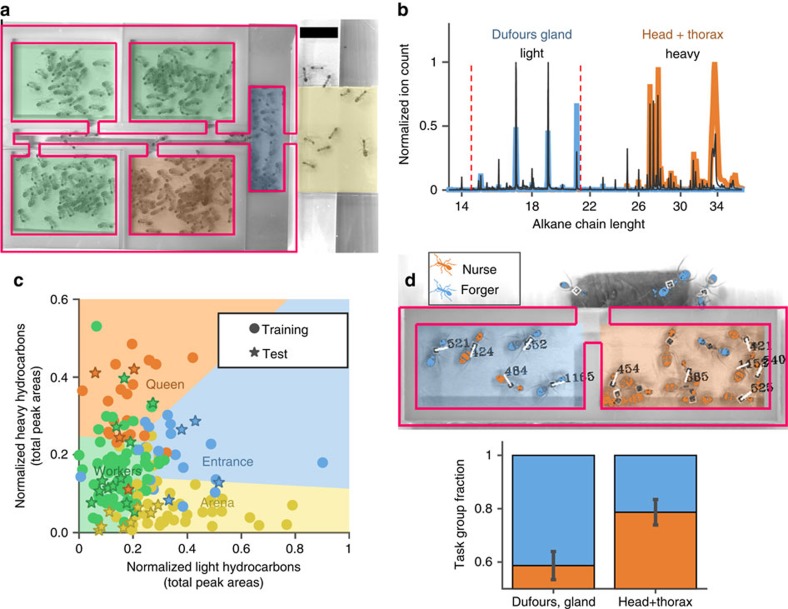
Chambers with a given function display a unique chemical signature. (**a**) Non symmetric nest with silica flooring. The borders of the nest structure are marked in magenta. Chamber colours correspond to: orange—queen's chamber, blue—entrance chamber, green—other inner chambers, yellow—arena. Scale bar (black) is 2.5 cm. (**b**) Raw chromatograms of nest floor extractions (gray), ant head and thorax extraction (orange) and Dufour's gland extraction (blue) reveals two distinct chromatogram regions with different physiological sources. Dashed red lines mark the borders of the defined ‘light' and ‘heavy' regions of the chromatogram. (**c**) Total ‘heavy' peak versus total ‘light' peak area of 133 training samples from 19 experiments (circles) and 28 test samples from 4 experiments (stars). The data point of each chamber is coloured according to its classification as in **a**. The training samples were used to construct a predictor with four classification zones which are here represented by the differently coloured polygons. The statistical significance of the classification is discussed in the following section titled ‘Accuracy of chamber classification' and quantified in Fig. 3. (**d**) Task group distribution in an artificially labelled nest. Nurses' head and thorax extraction was applied to the right chamber while foragers' Dufour's gland extraction was applied to the left one. The top image shows a snapshot of the barcoded ants' locations within the nest where nurse ants are marked in orange and forager ants in blue. The borders of the nest structure are marked in magenta. The width of the nest entrance is 1 cm. Bottom: bar-plots, employ the same colour code and signify nurse and forager distributions in *N*=5 binary choice experiments (error bars are s.d.).

We housed colonies (*N*=23) in five chamber asymmetrical nests (Fig. 2a) that enable a clear association between different chambers and the task groups that occupy them. Using the structure of the nest and the occupancy of the ants we classify the following locations: entrance chamber, queen's chamber, worker chambers and arena area. Worker chambers can be further divided into brood chambers and non-brood chambers (see Methods section ‘Nest structures') but the distinction between these is not as reliable since the number of brood items per chamber fluctuates and is, generally, difficult to count. Nest floors were lined with silica-covered glass to enable the measurement of minute amounts of chemicals left by the ants on these surfaces. After five days in the nest, ants were removed and the hexane extracts of the silica flooring of each chamber were separately analysed by gas chromatography (see Methods section ‘Extraction of surface chemicals'). We find that hydrocarbons of various lengths are the main components of the nest floor hexane extracts (Supplementary Note 5 for the full list). Similar to previous work^25^,^28^, we find the correlations between the abundancies of different hydrocarbon groups to be colony specific (Supplementary Note 6).

Here, we take the next refining step in which we associated specific chemical signatures not with different colonies but rather with chambers of different functionalities within the nest of a single colony. Comparing the samples extracted from the nest surfaces to those extracted directly from the ants (see Methods), we traced the sources of nest odours to different parts of the ant's body (Fig. 2b). Specifically, the lower molecular weight components (lighter than heneicosane (C_21_H_44_)) of the nest chemical signature such as heptadecane (C_17_H_36_), nonadecane (C_19_H_40_) and heneicosane (C_21_H_44_) are characteristic of the ants' Dufour's gland. The substances of higher molecular weight include a large number of compounds that are characteristic to this species' cuticular hydrocarbon profile^31^. Using this natural separation we set a mass threshold at heneicosane (C_21_H_44_) that divides the chromatogram to two parts: ‘light' (that is, relatively low molecular weight and low boiling point) and ‘heavy' (that is, relatively high molecular weight and high boiling point) compounds. Each chamber in each experiment was then attributed with two values which are the total area under the light and heavy sections of the chromatogram. Each of these values was separately normalized such that its sum over all internal chambers, in a specific experiment, adds up to one. This normalization allowed us to compare data points taken from colonies of different size and hence different amount of hydrocarbon content. Fig. 2c depicts the position of each chamber in the space spanned by these two normalized measurements. We find that areas of different function, that is, the entrance chamber, the queen's chamber and the worker chambers, as well as the arena fall in different regions of this plot.

We find further evidence that the classification of the chemical signatures of worker chambers may be refined into ‘brood' and ‘non-brood'. Indeed, the mean intensity of heavy compounds is 0.19±0.005 in brood chambers (*N*=28) and 0.11±0.01 in worker chambers (*N*=20). While these values are significantly different (*P*<0.01 two sided Kolmogorv–Smirnov test) we have not analysed this distinction further due to the difficulty of accurately quantifying brood numbers.

Taken together, these results imply that nest chambers that differ in their functional context also differ in their chemical signature. Conversely, the chemical signatures of chambers that serve the same function, despite apparent variability, are similar to a degree that could facilitate the recognition of their function. Moreover, establishing these differences neither requires the identification of each compound separately nor complex weighted sums over many compounds. Rather, chambers can be classified by the total density of only two large groups of compounds: the ‘light' and the ‘heavy' compounds. The next section discusses the statistical significance and accuracy of this simple classification approach.

### Accuracy of chamber classification

**Figure 3:**
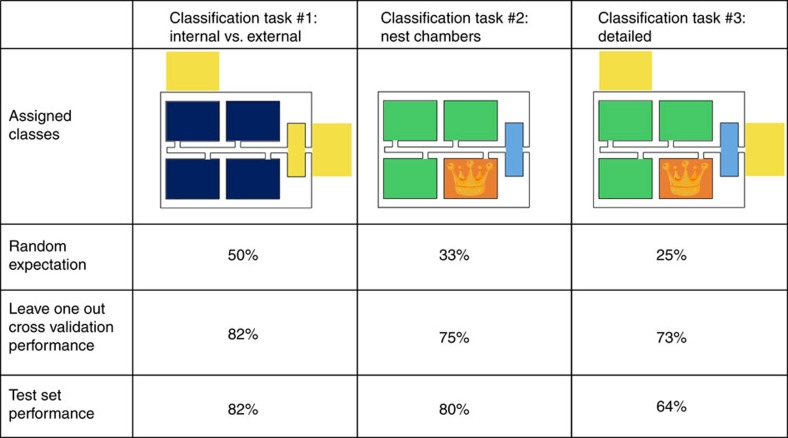
Classifier evaluation. Performance of three classification tasks that differ in their level of detail, appear in three columns, respectively. The first task corresponds to classifying internal chambers versus external chambers (that is, entrance and arena). The second task classifies three inner nest chambers (workers, queen and entrance chambers) while the third task aim to classify each of the four chamber types (workers, queen and entrance chambers and arena area). Results compare random expectation (one over the number of classes in a task) to the performance of leave-out-out cross validation over the training set, and to performance over an independent new test set.

We used supervised learning to construct classifiers that categorize each chamber using only two coarse-grained features: the total area under the heavy and light sections of the chromatograms. A training set (*N*=19 experiments including 133 samples) was used in three classification tasks that differ in their level of detail (Fig. 3). We employed a linear support vector machine^32^,^33^,^34^ (SVM, see Methods for further details) algorithm to construct a classifier for each of the three classification tasks. SVM is a supervised learning method which receives as input the labelled training data (Fig. 2c) and outputs optimal linear separators between pairs of classes, which are, in our case, chambers of different functionality.

An independent test set (*N*=4 experiments including 28 samples) was used to test the three classifiers determined through SVM. The performance was comparable to our cross-validation estimates, thus displaying the robustness of our classifiers and the effectiveness of our chromatogram-based features (Figs 2c and 3). The results suggest the presence of at least four different chemical signatures each of which eliciting a different behavioural response (Fig. 2c). Together with the characteristic signature of the flooring under the ants' refuse pile and the division of worker chambers into brood chambers and non-brood chambers, our results provide evidence for multiple road-signs that the ants may use within the nest.

### Manipulating task group positions through floor composition

We next verified that ants from different task groups differentially react to nest floor hydrocarbon composition. Specifically, we tested the prediction (generated by the SVM analysis) that forager ants will accumulate in chambers that are enriched in light hydrocarbons while nurses and queen's chamber ants will be accumulated in those enriched in heavier hydrocarbons.

To this effect, we used a simple nest structure in which ants entering the nest must choose one of the two symmetrically positioned chambers (Fig. 2d). The chambers' floors were artificially prepared such that each one of them displayed either a ‘light' or a ‘heavy' hydrocarbon blend. We allowed tagged foragers (*N*=15) and nurses (*N*=15) to enter and occupy the labelled nest (for further technical details see Methods section ‘Binary chamber choice experiment'). We then recorded the positions of the different ants during the first two hours after their introduction to the nest. We find that the fraction of nurses in the chamber that was labelled with head and thorax extraction was higher than their fraction in the chamber that was labelled with Dufour's gland extraction (*N*=5 experiments, *P*=0.015, one sided Wilcoxon signed rank test, Fig. 2d). The converse holds for foragers since they make up for all the remaining ants in this test. Control experiments, in which only one of the chambers was labelled, rule out the possibility that ants react to only one of these two signatures (Supplementary Note 13). These results imply that nurses can generally be associated with the ‘heavy' labelled chamber and foragers with the ‘light' labelled chamber as predicted by our statistical analysis (Figs 2c and 3).

To summarize, our results provide behavioural evidence that the ants react to at least three of the identified signatures: the queen's chamber, the entrance chamber and the refuse area.

### Identifying crucial compounds for chamber classification

In general, we found that areas populated mainly by foragers are characterized by higher proportions of light hydrocarbons that originate from the ants' Dufour's gland. Indeed, chemicals originating from this gland have previously been associated with the ants' foraging activity^35^,^36^,^37^,^38^,^39^. Although Dufour's gland extracts are, to an extent, colony specific their composition across colonies predominantly includes, light section hydrocarbons of 11–21 carbons^40^. Specifically, for all colonies, the main chemical components of the light section were: heptadecane (C_17_H_36_), nonadecane (C_19_H_40_) and heneicosane (C_21_H_44_). The small number of compounds in the light compound group and the low signal collected from each dictated that, to obtain reasonable classification, we had to use the total sum over the all light compounds.

In contrast, the queen's chamber displayed a strong signature of heavy hydrocarbons as characteristic of ants' cuticular profile (Fig. 2c). Contrary to the few light compounds that were identified in the light section of the chromatogram, the heavy section includes tens of compounds. To pinpoint those heavy compounds that are sufficient for queen chamber classification we repeated the learning procedure described above using arbitrary subsets of compounds. Specifically, instead of using all heavy compounds we used only a certain subset of them. Many such subsets were found to support accurate chamber classification. Examples for such compounds include a mixture of 11- and 13-methyl-hentriacontane and a mixture of 11,15- and 13,17-dimethyl-hentriacontane all of which are characteristic *C. fellah* cuticular hydrocarbons^31^,^41^. We find that any such characteristic compounds exhibits a strong correlation with the total amount of all other heavy compounds (Supplementary Note 11). This suggests a high redundancy in the chemical signature: ants may either be reacting to certain, specific substances from this list or to the sum of all heavy substances.

### Origin of chemical signatures

It was previously shown that ants from different task groups exhibit different chemical profiles on their cuticles^42^,^43^ and have tendencies to spatially cluster in specific chambers within the nest^44^. This suggests a simple passive mechanism for the non-homogeneous distribution of chemicals within the nest: the chemicals on the ants' cuticles rub off onto the surfaces of chambers which they inhabit such that the spatial fidelity of different task groups results in a non-homogeneous distribution of chemicals. A purely passive marking mechanism would imply that the signatures found on the nest floors coincide, to a large degree, with the hydrocarbon profiles as measured directly from the ants' cuticles. In this section we provide evidence that suggests that even if this passive mechanism is at work then it must be supplemented by additional processes.

**Figure 4:**
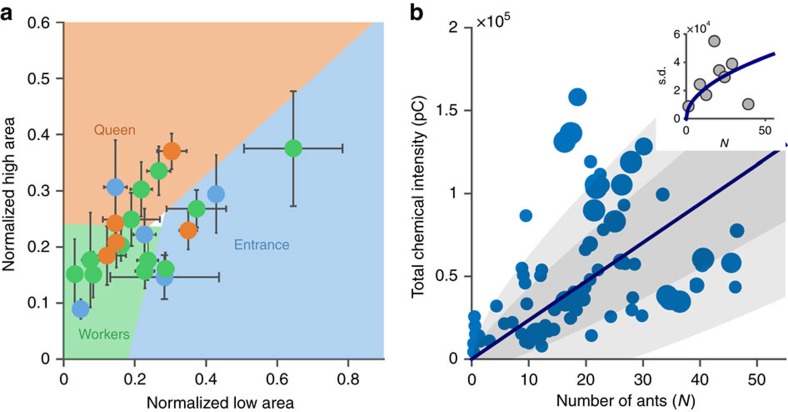
Evidence against passive chemical deposit mechanism. (**a**) Values of the ‘light' and ‘heavy' variables as calculated by taking the mean over groups of five ants that were collected from given chambers (circles) overlaid on the chambers' classification regions identical to those presented in Fig. 2b (*N*=5 experiments, error bars are s.d.). The colour scheme for both the ant and the chamber data are as marked on the figure. (**b**) Total peak areas as measured from floors of chambers with different number of ants (as averaged over 5 days of experiment). The solid line is a linear fit with a slope *m*_ant_=2.3 × 10^3^ which indicates the mean amount of pheromone passively left by an individual ant. The dark and light gray areas respectively mark distances of one and two standard deviation from this fit. Marker size reflects the relative time the queen has stayed in each chamber. Inset shows the s.d. of the binned data versus the number of ants in the chamber. In a linear model, one would expect this fit to obey 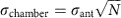 where *N* is the number of ants in the chamber and *σ*_ant_ denotes the s.d. in the amount of chemicals passively left by a single ant. Fitting this function to the measured points yields a value of *σ*_ant_≈3*m*_ant_.

First, as shown above, the light compounds, which appear on the floorings of all chambers, appear on the ants' cuticles in minute amounts only (Fig. 2b). Rather, we have traced these compounds to the ants' Dufour's gland (Fig. 2b)^40^. Second, the chemical samples we extracted directly from the ants' cuticles (in five of the 23 experiments) did not match those present on the surfaces of the chambers these ants occupy. In other words, the chemical classification method, successfully used to identify the floors of different chambers, failed to segregate between the extracts of ants collected from different chambers (Fig. 4a). Likewise, although queens are known to have a specific odour^45^,^46^, queens' extracts did not fall within the queen chamber classification region. This excludes a passive mechanism involving queen-specific chemical signatures (for more details see Supplementary Note 8). In addition, differences in the hydrocarbon characteristics of worker chambers with or without brood cannot be explained by simple shedding of chemicals off of brood cuticles since direct measurements of the brood cuticular hydrocarbons displayed negligible signals (Supplementary Note 9). Finally, a completely passive transfer mechanism from the ants' cuticles to the nest surfaces would imply a simple linear relation between the total mass of chemicals extracted from each chamber and the number of ants that occupy it. Although a positive correlation between these two variables exists (Fig. 4b), noise is substantial. This variation is somewhat larger in chambers that are associated with queens (large dots in Fig. 4b) than in worker chambers (smaller dots in the same figure). This noise cannot be attributed to the much smaller sampling errors of ant numbers, surface chemical measurement noise (Supplementary Methods), ant to ant variation in size (see section ‘Video and barcoding'), evaporation of chemicals from the surface (see section ‘Extraction of surface chemicals') or ant to ant variation in total cuticular chemical content (see section ‘Direct chemical extraction from ants'). The observed high levels of overall noise can occur only if the standard deviation of the amount of chemicals left by each ant is about three times larger than the mean amount per ant. This indicates that a significant fraction of the ants have negligible contribution to the chemical intensity while another fraction has a large, above average contribution. These large variations provide another piece of indirect evidence against the exclusivity of passive chemical transfer, especially in the queen's chamber.

Taken together, these results support the claim that a simple mechanism in which the chemical hydrocarbon profile of a certain task group would also characterize the chamber in which this group resides does not suffice for explaining our results. Rather, other mechanisms seem to be at play. It is, however, worth noting that even active secretion is not in itself sufficient proof that the secreted chemicals serve as pheromonal signals that the ants have specifically evolved to differentially mark the different areas of the nest^47^. Testing whether the chemical signatures of the nest consist of cues that are a byproduct of the ants' presence in the room or, rather, complimented by actively generated signals is the subject of future research.

## Discussion

Social insects are fascinating because they manage to coordinate their actions without central control. In the ant colony this coordination is tightly linked to the ants' spatial distribution. Combining a methodology for detecting chemicals adsorbed onto the nest surfaces with individual ant tracking techniques and machine learning, we have revealed that ants utilize complex chemical patterning within the nest. These chemical signatures constitute a new form of stigmergy and serve as ‘road-signs' that assist ant orientation, colony stabilization and spatial segregation between different task groups.

Our work provides the first experimental evidence of a navigation mechanism of any possible type that aids ant orientation within the nest. While it was previously known that ant hydrocarbons are found on nest surfaces^25^,^27^,^28^, their function, if any, remained unclear. Here we show that chemical signatures assist navigation within the nest. We identify six different chemical profiles that signify different chamber functions within and around the nest (queen's chamber, worker chambers with or without brood, entrance chamber, refuse area and proximal foraging arena) and more probably exist. Moreover, we present direct behavioural evidence that the ants recognize and follow at least three of these road-signs into specific nest areas. Despite the richness of these chemical road-signs we hypothesize that this stigmergic mechanism is not exclusive and complemented by other subterranean navigational tools such as spatial memory and quorum sensing.

The description of the social insect colony as a superorganism (or social-organism^48^) has proven to be a useful analogy of exceptional breadth^49^. Our work suggests the addition of yet another layer to this correspondence by relating the nest chambers to different organs, and the ants to migrating cells^50^,^51^,^52^. Using the principles of stigmergy, passively or actively generated chemicals serve as the superorganism's chemokynes^53^. Multiple chemical signals serve to differentially guide and stabilize trafficking and organization of the mobile agents that make up the whole. Future work will be required to further explore the validity and usefulness of this compelling analogy.

## Methods

### Ants

A total of 15 queenright *C. fellah* colonies containing 60–110 workers were used for the experiments. The colonies were reared in the lab from mated queens collected at the Weizmann Institute campus in Israel between 2011 and 2014. The colonies were kept in a climate-controlled chamber under controlled humidity (65%), temperature (27 °C) and a light dark cycle of 12 h. Ants were supplied weekly with a food mixture containing tuna, honey, eggs and a vitamin mix, and water *ad libitum*.

### Nest structures

Structural data of *C. fellah* nests is unavailable. The tendency of this species to build its nests intertwined with tree roots make it difficult to cast. Choosing the number and size of chambers of the artificial nests we referred to the known nest structure of *Camponotus socius,* a related species^54^ which contains 2–10 chambers with a spatial dimension of several centimeters.

Symmetric four chambered nests were constructed by dividing a 15 cm petri dish into four identical chambers accessible from a common 1.5 cm square entrance cut in the center of the petri dish cover (Fig. 1a–e). Symmetric two chambered nests were constructed by cutting two identical 2.5 × 5 cm^2^ chambers in a 11.5 × 3.5 × 0.4 cm^3^ Perspex plate accessible from a common 1 cm entrance (Fig. 4d). In these symmetric nests the entrance is located at equal distances from each of the chambers so that the ants' chamber choice will not be influenced by the nest geometry.

Non symmetric nests were constructed by cutting four 4 × 5 cm^2^ internal chambers and a 2 × 5 cm^2^ entrance chamber in a 13 × 17 × 0.4 cm^3^ Teflon plate. The nest structure was cleaned using ethyl acetate, hexane, and acetone. Cleaned silica plates placed on a filter paper served as the nest floor, and IR filters were used as the nest top. Two more silica plates were placed outside the nest to measure chemicals deposited on the foraging arena floor.

All nest structures were designed to prevent the penetration of visible light. Visible light illumination was used as a means of encouraging the ants to emigrate into the dark nest. Experiments themselves were done under IR illumination (Exolight, Metaphase, 850 nm). In order to allow filming the top of the nest was covered with an IR transparent filter and a camera (for camera details see section ‘Video and barcoding‘) with no IR blocking filter was used. The experimental setup was surrounded with black curtains to block visible light. In all experiments the nest structures were placed in a 30 × 20 cm^2^ glass baking pan (Pyrex) with fluon (Sorpol) coated walls to prevent ants from escaping.

Asymmetrical nest structures induce a clear association between different chambers and the task groups that occupy them. We define the following areas in and around the nest: Arena—areas around the nest; Entrance—the chamber closest to the door, this chamber consists mostly of lean forager ants (Supplementary Methods); Queen's chamber –the chamber in which the queen had spent over 50% of the time (such a chamber occurred in all experiments); Worker chambers—any chambers which did not fall into any of the previous categories. Queen and worker chambers were occupied by more corpulent individuals (Supplementary Methods).

Worker chambers could be further refined into brood chambers and non-brood chambers. The brood content of worker rooms was estimated by manually counting brood items in ten video frames of each experiment. Worker chambers in which the mean number of brood items was less than one were labelled non-brood chambers, other worker chambers were labelled as brood chambers. A mean number of 1.9474±0.8481 chambers out of a total of three worker chambers per experiment were defined as brood related.

It is important to say that although the asymmetrical structure promoted the segregation of task groups into different nest chambers this does not mean that a specific chamber (say the leftmost top one as in Fig. 2a) occupied the same task group in all 23 experiments.

### Nest surfaces manipulation

Colonies were housed in symmetric four chamber nests with paper floors for a five-day ‘priming stage' after which the paper floors were transferred to a new identical nest and the colony was allowed to move into the new nest structure. This stage was performed after removing the queen from the colony to avoid the large stochastic effects that may be induced by this single individual. Shuffling the floor segments ensures that the ants could not rely on individual memory when inhabiting the new nest. The precise symmetry between the different chambers similarly ensured that ants could not identify specific locations by virtue of chamber structure. As an extra precaution, we further prevented the use of any possible visual signals by running the experiment under infrared lighting^55^,^56^. These measures guarantee that if the ants are able to return to their original locations within the nest then this could only be attributed to properties of the paper floors that had been acquired during the priming period due to the ants' presence. The ants' locations within the nest were compared in two three-hour periods, one just before the manipulation and the other 21–24 h after the introduction of the new nest. The second time period was set to coincide with the earliest time at which most ants in all experiments had entered and reoccupied the nest.

### Silica floor preparation

Silica on glass thin layer chromatography (TLC) plates (Analtech) were cut to 6 × 5 or 3 × 5 cm^2^ to fit the size of the nest chambers so that separate chambers did not share the same floor tile. This was done to prevent leakage of materials between chambers through diffusion within the silica layer. Before being positioned as chamber floors, the plates were thoroughly cleaned using ethyl acetate, hexane and acetone (For further details regarding the cleaning procedures see Supplementary Methods).

### Video and barcoding

Ants were tagged with 1.9 mm^2^ stamps printed with 6 × 6 2D barcodes (BugTag, Robiotec). Tags were attached to the ants' dorsal thorax using a small amount of skin adhesive^7^ (original Sauer skin adhesive, Manfred Sauer). Experiments were imaged from above using four 8MP cameras (JAI, ab-800cl) with a camera link connection to a frame grabber (Matrox radient eCL) such the internal parts of the nest were filmed through the IR filter. Each camera filmed an area of 13 × 17 cm^2^ to allow sufficient resolution for barcode identification. Barcode labelled ants were identified by a commercial computer vision-based tracking system (BugTag, Robiotec) in real time at 8 Hz.

In the experiments presented in section ‘Classifying chamber function by its chemical signature' the location of the queen and the number of ants per chamber were automatically recorded once every 5 min during five days. These experiments did not require the identities of individual ants and the location of individual (but unnamed) ants was tracked by blob analysis using the MATLAB image analysis toolbox. We find that workers tend to occupy the same location for long periods of time and therefore the mean is a good estimation of the number of ants in this chamber at any particular point in time. The number of ants in a specific chamber does not change significantly over the course of the measurements and the variations are on the order of 10% of the mean. The errors of this automatic counting algorithm were assessed by comparing it to manual counts over 10 frames. We quantified the counting error for a single frame at 16% which makes the error in estimating the average over 1,440 frames (the quantity used in Fig. 4b) as low as 16%/√1440. In addition to this, performing the same analysis using the total pixel area of all ants instead of their number yields similar results.

### Permutation test

A Permutation analysis was used to test the significance of the relation between floor segments and ant locations. The contour plots as presented in Fig. 1a–d were projected onto four component vectors 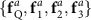 that indicate, for each ant *a*, the fraction of time spent on each of the four floor segments before the manipulation (Q indicates the floor segment of the chamber in which the queen resided for the majority of her time). During the manipulation, the segments were removed from the original nest and inserted into a clean nest structure such that ∑ (*j*)=*k*, where ∑ is a one-to-one function (permutation) associating each of the four segments 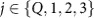 to its shuffled location in the new nest 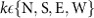. The ants then reoccupied the new nest and the fraction of time each ant, *a*, spends in each of the four chambers is denoted by: 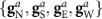.

Computationally, we define a score *S*^*σ*^ for each possible one-to-one assignment function *σ*:{Q,1,2,3}→{N,S,E,W} as:


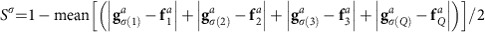


where the mean is taken over all ants, *a*, in the colony. This measure varies between 0 and 1. If it occurs that each ant returned to exactly the same floor segments after the manipulation as she had occupied before it (including fraction of time spent) then the actual experimental assignment *σ*=∑ will be scored as *S*^^σ^^=1. If ants tend to return to floor segments that are independent of their locations in the first stage of the experiment then the distances *S*^*σ*^ can be expected to be closer to the minimal value of 0 for all possible assignments *σ* (including the realized assignment *σ*=∑). In the non-ideal case in which the floor segments do contain imperfect orientational information which the ants only partially follow, one would expect higher scores *S*^*σ*^ for permutations *σ* which are more similar to the experimental chosen permutation, ∑. We rank permutations by their score.

### Extraction of surface chemicals

To identify which hexane soluble compounds appear in which chambers requires measurements of the levels of different chemicals from the surfaces within the nest. This is not trivial because the large volume of the surface together with the minute amount of chemicals left by the ants can result in low signal-to-noise ratios (SNR).We devised a method that utilizes silica covered glass floorings (thin layer chromatography TLC plates, Sigma) which have low background signal (Supplementary Note 12) and can accumulate the signal, due to the high porosity of the silica powder, and thus enhance the SNR (for details, see Supplementary Methods). We further reduced noise to sufficiently low levels by scraping the silica powder off of the glass support plate prior to hexane extraction. We verified that this method yields high SNR, high reproducibility, and a linear relation between analyte mass and GC output signal (see Supplementary Methods and sections ‘GC-FID analysis' and ‘GC–MS analysis' below).

Specifically, silica powder was scraped off the glass support and placed in glass vials to which 1.5 ml of hexane were added. Each vial contained the silica from the full floor of a single chamber. In the case where a refuse pile was created within the nest (seven out of the 23 experiments presented in section ‘Classifying chamber function by its chemical signature'), the silica from the flooring underneath this pile was collected into a separate vial. The vials were sonicated for 20 min to increase yield. The resulting supernatant was transferred to clean vials. This procedure was repeated to maximize the extraction yield. Excess solvent was evaporated under a nitrogen stream to a total volume of 100 μl out of which 50 μl were analysed by gas chromatography (either GC-FID or GC–MS).

The majority constituents (by mass) of the nest floor samples are heavy hydrocarbons (>heneicosane, C_21_H_44_) which are solid at room temperature. It is therefore likely to assume that the overall mass of nest floor samples does not change significantly due to evaporation.

### Direct chemical extraction from ants

In five out of the 23 experiments presented in section ‘Classifying chamber function by its chemical signature' we sampled five ants from each chamber for total hydrocarbon analyses. Individual ant cuticular hydrocarbons were extracted using silica powder so as to imitate a passive process by which ants rub their cuticular hydrocarbons on the silica floorings unintentionally (A similar extraction method is described in ref. 57). Ants were placed in 2 ml glass vials containing 0.5 g of cleaned silica powder (Analtech). The vials were inverted several times during one minute to increase the contact area between the ants and the silica such that a thin layer of cuticular hydrocarbons will be removed from their entire body. The resulting silica extracts were prepared for GC analysis as described above. After this treatment, the ants were removed from the vials and dipped in water to remove any excess silica powder from their bodies. This wash allows 100% of the ants to survive the extraction procedure.

Comparing the cuticular hydrocarbon profiles extracted from individual workers we find that the standard deviation which represents the variability between the total intensity of chemicals extracted from each ant is 80% of the mean intensity over all ants. A possible source of variability is the size of the ant (and, correspondingly, the overall area of her cuticle). This number is an overestimation of the variability as it includes the measurement error.

For the data presented in Fig. 4a, individual ant samples were normalized by taking the mean over samples collected from ants who resided in the same chamber (*N*=5) such that each ant group from each of the chambers was attributed ‘light' and ‘heavy' values. Each experiment was then normalized such that the ‘light' and ‘heavy' values summed to one separately, in the same way nest floor samples were normalized.

For the preparation of the head and thorax and Dufour's gland extractions, 10 workers from the same colony were killed by freezing and then immediately dissected. The head and thorax were separated from the abdomen and placed in 1 ml hexane. The Dufour's gland were excised by dissection and placed intact in 200 μl hexane. The hexane solutions were transferred to new vials 24 h after the beginning of the extraction.

### GC-FID analysis

All samples were analysed using large volume injection methods^58^ in order to increase sensitivity. Samples (50 μl) were analysed on a 7890 Agilent gas chromatograph equipped with a fused silica column (DB5-MS 30 m × 0.25 mm × 0.25 μm, Agilent) and coupled to an FID. Inlet temperature was set to 30^o^c and vent flow was set to 100 ml min^−1^ for 1.02 min at 5 PSI after which the inlet was heated to 325 °C at 600 °C min^−1^. The oven program started at 30 °C where it remained for 3.52 min, raised to 270 °C at 10^o^c min^−1^ where it remained for 5 min and raised to 310 °C at 30 °C min^−1^ where it was held for 15 min. The instrument was operated at constant flow of 2 ml min^−1^ and the detector temperature was set to 300 °C.

### GC–MS analysis

All samples were analysed using large volume injection methods^58^ in order to increase sensitivity. Samples (50 μl) were analysed on a 7890 Agilent gas chromatograph coupled to a LECO Pegasus time of flight mass spectrometer equipped with a Gerstel cooled injection inlet and a fused silica column (DB5-MS 30 m × 0.25 mm × 0.25 μm, Agilent). Inlet temperature was set to −21^o^c, the vent flow was set to 260 ml min^−1^ at 7.5 PSI and the injection speed was set to 1.04 μl s^−1^. The inlet temperature was kept for 1 min after which it was heated to 260 °C at 720 °C min^−1^. The oven program was initiated at 30 °C where it remained for 3 min after which it was raised to 310 °C at 8 °C min^−1^ where it remained for 10 min. The instrument was operated at constant flow of 1 ml min^−1^. The various compounds were identified by their mass fragmentation.

### Chemical classification

Classification procedures were based on a training set of 19 experiments containing 7 samples each. A class was assigned to each sample in the following manner: two samples collected from plates placed outside of the nest structure; one sample collected from the entrance chamber; one sample collected from the queen's chamber; and three samples collected from the three workers chambers. For a definition of these categories see the ‘Nest structures' section of the Methods. Each sample was represented by the sum of its light/heavy compounds, that is, the feature space is 2D and classification is performed in the 2D plane. Three classification tasks, having different levels of detail, were considered: (1) classification into two classes: internal nest chambers versus external chambers (entrance and arena), (2) classification into three classes corresponding to the three inner nest chambers (workers, queen and entrance chambers) and (3) classification into four classes: A detailed classification of workers, queen, entrance chambers and arena area.

Classification was based on a linear support vector machine (SVM). We used a linear SVM procedure that provides separation between two classes only. In order to classify more than two classes we conducted a majority vote over SVM classifiers between all pairs of classes. For example, in the third classification task, this procedure bounds the area signifying the queen's chamber signature (Fig. 2c) by the line separators obtained from three (queen versus workers, queen versus arena and queen versus entrance) independent SVM classifications (as conducted on the training set). Results were evaluated using leave-one-out cross validations, that is, each 18 out of the 19 experiments were used for training and then tested on the remaining experiment (results correspond to the average classification accuracy over the 19th sample). This procedure allowed us to produce, for each of classification tasks, a classifier that optimally separates the samples from the 19 training experiments. Classifier performances were then evaluated according to their success in classifying a completely disjoint test set containing 28 samples from four newly performed experiments. The analysis was done using MATLAB statistics and machine learning toolbox.

### Statistical analysis

The statistical significance of the results presented throughout the text was evaluated, in most cases, using standard statistical analysis. The names of the tests that were used are given near each such statistical statement.

In some cases, *P* values were calculated as the weight of the tail of a binomial distribution in which we use the null-hypothesis which states that the tested factor has no effect. This can be formulated as the probability of n or more successes during *N* trials: 
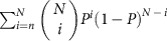
 where p is the probability of success in a single trial under the null-hypothesis. For example, in section 'Ants have preferred locations within the nest‘ we calculated the probability that ants which leave the nest (*N*=40 ants in 6 colonies) would return to the same chamber they exited and found that 17 out of 40 ants returned to the same chamber. To assess the statistical significance of this result we used a null-hypothesis which states that all floor segments are identical and that the probability to return to a specific segment is *P*=0.25 (as there are 4 chambers). The *P*-value was calculated by the formula presented above. In all experiments in which we had control over sample size, this was chosen using power analyses (using MATLAB software) with power of 0.8 when compared with an alternative hypothesis H1 with *P*=0.99.

### Binary chamber choice experiment

As stated in section ‘Manipulating task group positions through floor composition' in the Results we verified that ants from different task groups differentially react to nest floor hydrocarbon composition using a double chamber nest. The chambers' floors were artificially prepared such that each one of them displayed a different hydrocarbon blend. This was done by collecting hexane extractions from (i) the ants' head and thorax, which mainly contain hydrocarbons from the ‘heavy' group, or from (ii) their dissected Dufour's glands, which mainly contain hydrocarbons from the ‘light' group (Fig. 2b).

Nurses' head and thorax extraction was prepared from 10 nurses in 1,500 μl hexane. Foragers' Dufour's gland extraction was prepared from 10 foragers in 750 μl hexane. Both extractions were prepared as described in section ‘Chemical extraction'. Extracts were subsequently analysed using a GC–MS and were found to contain the same compounds as nest floor samples (Fig. 2b), mostly hydrocarbons. The extracts' total hydrocarbon concentrations were assessed by GC-FID analysis to be ∼5 ng μl^−1^ for the head and thorax extraction and ∼10 ng μl^−1^ for the Dufour's gland extraction. Symmetrical two chamber nest structures (Fig. 4d) were prepared as described in ‘Nest structures' section. Foragers and nurses (15 of each group) that were used as test ants were collected and tagged as described in section ‘Video and barcoding'. To prevent repellence that could be elicited by the exposure to odours of conspecific colonies^59^, the test ants were collected from that same colony that was used to prepare the extractions. Foragers were identified as ants that were found outside the nest, nurses and queen related workers were identified as ants that were found inside the nest, in the near vicinity of the brood pile. An artificially labelled nest was prepared by applying 24 μl nurses' head and thorax extraction in one chamber and 12 μl foragers' Dufour's gland extraction in the other chamber. The concentrations were chosen such that they coincide with those of naturally occurring nest hydrocarbons as revealed by our measurements (The densities of the nest floor samples were estimated to have a median value of 5 ng cm^−2^ of total chemical intensity per unit area. The density chosen for the described experiments was 10 ng cm^−2^). Each nest was used only once. The extractions were applied directly onto the Perspex floor as homogeneously as possible attempting to create uniform coating. The relative location of each extract and position of the nest structure were randomized so that consecutive experiments would be independent. An experiment was initiated by placing a labelled nest in a 30 × 20 cm^2^ glass baking pan (Pyrex) containing the tagged subgroups of workers under visible light illumination. Experiments were filmed for two hours after the introduction of the labelled nest as described in ‘Video and barcoding'.

### Code availability

Computer codes that were used to generate the data presented in this study are available from the corresponding author upon reasonable request.

### Data availability

The data that support the findings of this study are available from the corresponding author upon reasonable request.

## Additional information

**How to cite this article:** Heyman, Y. *et al*. Ants regulate colony spatial organization using multiple chemical road-signs. *Nat. Commun.* **8,** 15414 doi: 10.1038/ncomms15414 (2017).

**Publisher's note:** Springer Nature remains neutral with regard to jurisdictional claims in published maps and institutional affiliations.

## Supplementary Material

Supplementary Information Supplementary Methods, Supplementary Figures, Supplementary Tables and Supplementary Notes

Supplementary Video 1 Nest surfaces affect ant spatial organization. This movie shows the behavioral manipulation done to show that ants recognize chemical road-signs inside the nest.
