# Supplementary material for: Ants regulate colony spatial organization using multiple chemical road-signs
Source: Nat Commun. 2017 Jun 1;8:15414. doi: 10.1038/ncomms15414 (PMC5461491; doi:10.1038/ncomms15414)
Supplement: Supplementary Information — Supplementary Methods, Supplementary Figures, Supplementary Tables and Supplementary Notes [file ncomms15414-s1.pdf]

Supplementary Methods

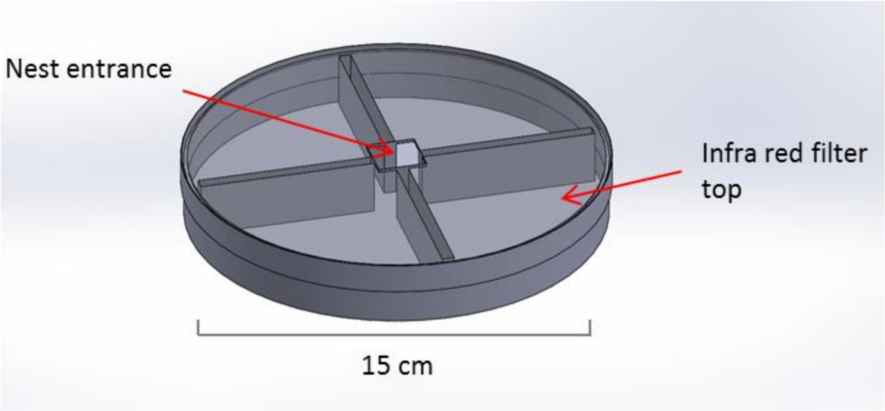

Supplementary Figure 1: Symmetric nest structure scheme. The nest is constructed from a 15 cm petri dish divided into 4 identical rooms. Nest top is covered with an infra-red filter which appears transparent in the scheme for visualization. Nest entrance is located at the top as indicated by a red arrow.

Permutation test

We show that the behavioral experiments presented in section *"Nest surfaces affect ant spatial organization"* support the presence of two different stimuli that are associated with the nest surfaces and affect the way in which ants divide between the chambers of the nest. These were six different experiments conducted on three different colonies. We do this by showing that cases in

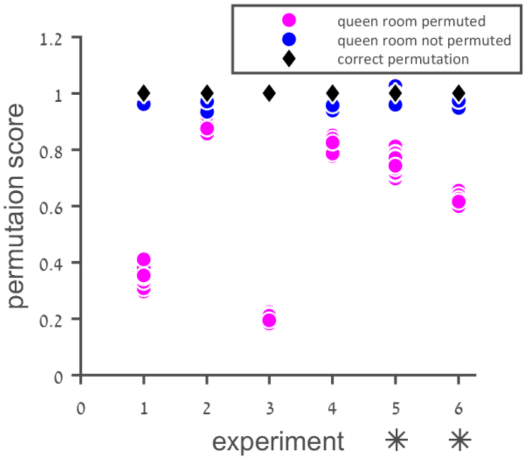

Supplementary Figure 2: shuffle analysis results permutation scores for the permutation test used to assess the correlation between the ant's locations in the period before they were removed from the nest and the period after they were reintroduced to the nest. The score of the correct permutation was subtracted from the rest of the scores in the experiment. The correct permutation is labeled with a black diamond, permutations in which the queen's chamber was not permuted are labeled blue circles and the rest of the permutations are labeled pink circles. Experiments in which the floor segments were randomly shuffled are marked with an asterisk. Unlabeled experiments were rotated by 90 degrees relative to their previous orientation.

which fewer stimuli are present are not statistically likely. We first test the null hypothesis that none of the chamber surfaces obtained any special characteristics during the priming period. Under this assumption, floor segments cannot be distinguished by the ants (see "*Permutation test*" in the Methods). Therefore, using the ants' locations to generate a guess regarding which of the four new nest locations {N,S,E,W} contains the Q floor segment (*i.e.* the floor segment on which the queen resided before the manipulation) would only succeed with a chance of 25%. The chance that the correct assignment is achieved over six independent trials would be $p = \frac{1}{4^6} < 3e - 4$ (weight of the tail of the corresponding Binomial distribution). Our experimental measurements enables locating the chamber which contains the Q floor segment in six out of six trials. This procedure that yields this is simple: of the 24 possible permutations, the six top scoring permutations were those in which chamber Q was strongly associated with a single location in the nest (this includes all six permutations in five experiments and four out of six permutations in one experiment). In all six experiments, the Q floor segment was indeed shuffled into this location. Again, the chances for this to happen randomly are very small.

In fact, one can reject the null hypothesis with even higher statistical significance. In all six experiments, among all possible permutations, σ, the one that corresponded to the actual experimental permutation, ∑, ranked within the top 12.5% (SI figure 2, "*Permutation test*" in the Methods). If the probability of returning to a chamber was completely independent of floor characteristic then the actual experimental permutation would be no different than any other randomly generated permutation. In this case, the chances that the correct association appears in the top 12.5% (or 1/8) of all permutations would simply be 1/8. The probability that this would happen by chance six time is, therefore, $\mathrm{p} = \frac{1}{8^6} < 4\mathrm{e} - 6$. We can, therefore, reject the null hypothesis that ants do not rely on floor characteristics when they return to the nest.

The previous results provide strong support for the fact that at least one type of stimulus, obtained during the priming period, affects the ants' spatial distribution in the nest after the manipulation. Next we show that, given our experimental results, a single stimulus is also unlikely. To do this, we assume four hypothetical situations and then refute them one by one.

- The first case is the one in which **the same single stimulus is present on all four floor segments**. In this case, the segments are indistinguishable from each other. This is

equivalent to the case of no stimuli at all which we have already negated in the previous paragraphs with $p < 4e-6$.

- The second case we consider is the one in which **a single stimulus is present on two of the floor segments**. In this case, there are two pairs of floor segments: one pair contains the stimulus and the other does not. By the null hypothesis, the two floor segments belonging to the same pair are indistinguishable by the ants (otherwise, there would have been two distinct stimuli in contrast to our assumption). We focus on the pair which contains the floor segment corresponding to chamber Q. Assume, without loss of generality, that these are floor segments {Q,1} and that they were inserted into locations {N,W}. We expect that after the manipulation these two segments would be equivalent from the point of view of the colony. In other words, the spatial distribution of ants after the manipulation could not hold any information regarding which of two chambers {N,W} contains floor segment Q. Any assignment thus has 50% chance of being correct. However, as shown above, in six out of six times it is actually possible to correctly accurately identify the nest chamber that contains the queen's floor segment Q. The probability that this happen, if the null hypothesis holds, is $\mathrm{p} = \frac{1}{2^6} < 2\mathrm{e}-2$ (weight of the tail of the corresponding Binomial distribution).
- The third case we consider is the one in which **only one floor segment (Q) contains a stimulus** while all other chambers contain no stimulus. To verify that the queen's chamber is not the only source of order in the system we removed the corresponding floor segment (and its location in the new nest – without loss of generality we name it E) from the analysis and repeated the permutation procedure described above. The goal is now to associate floor segments {1,2,3} with their locations in the new nest {N,S,W}. Again, we find that the permutation, σ, which is consistent with the experimental manipulation, ∑, ranks high (see *"Permutation test"* in the Methods) .In fact, it is ranked highest in four cases, and second and third in one case each. Similar to the calculation in the previous paragraph the probability that such high ranking would be achieved at random given the null hypothesis of just one floor segment containing a stimulus can be computed by adding the probabilities to reach these ranks or a better combination of ranks. Specifically, we calculate the probabilities to reach the combinations of ranks shown in SI table 1.

**Rank order (out of 6 possible permutations)**						**Number of possibilities**
1	***2***	***3***	***4***	***5***	***6***	
4	1	1	0	0	0	$2\binom{6}{4}$
4	2	0	0	0	0	$\binom{6}{4}$
5	0	1	0	0	0	$\binom{6}{5}$
5	1	0	0	0	0	$\binom{6}{5}$
6	0	0	0	0	0	1

Supplementary Table 1: Rank combinations that are either as good as the rank combination received in the experiments or better.

Summing the counts in table SI 3 and normalizing by the number of possible experimental outcomes (6^6) shown in SI table 3 we calculate the p-value to be $p = \frac{\left(6+6+1+\binom{6}{4}(1+2)\right)}{6^6} = 1.2e-3$.

- The final possibility is the one in which **one stimulus exists and is present in three out of the four floor segments**. In this case the queen's segment must be the only one that does not contain the stimulus while the other three chambers are indistinguishable. This is precisely equivalent to the case of one stimulus in a single chamber, a case which was refuted above.

To summarize our results show that the null hypothesis stating that no information is transferred in the floor segments can be rejected with probability $p = \frac{1}{8^6} < 4e-6$. Moreover, the second null hypothesis that a single type of stimulus is present on some or all of the floor segments can also be rejected. A p-value for each possible case was presented above and the highest of these is p < 2e-2. Therefore, our results support the fact that, following the priming period, at least two stimuli are present in the floor segments that are distinguishable by the ants and affect their final distribution in the new nest.

Extraction and analysis method performance

We used a linear hydrocarbon mix to assess the SNR, reproducibility, and linearity of this method (SI figure 5). Samples were analyzed on a 7890 Agilent gas chromatograph equipped with a fused silica column (DB5-MS 30 m × 0.25 mm × 0.25 µm, Agilent) and coupled to an FID as described in section *"GC-FID analysis"* in the Methods. The SNR, per chromatogram peak, was defined as the ratio between the measurements' mean value to the blank signal mean value (taken over all relevant peaks). We find a mean SNR of 1.4e5 for 6 ng of standard weight on a surface of 20 cm^2 (SI figure 5a). Reproducibility was quantified by calculating the coefficient of variation (standard deviation divided by the mean) over three replicate measurements (SI figure 5b). As an example, for 6 ng of standard weight on a surface of 20 cm^2 for alkanes with 15 carbons or more yields ($N=25$) a median coefficient of variation of 0.065. Finally, our measurements exhibit a linear relation between the amount of analytes and the output GC signal (SI figure 5c) this relation persists over a wide range of total injected mass (low discrimination, SI figure 5c inset).

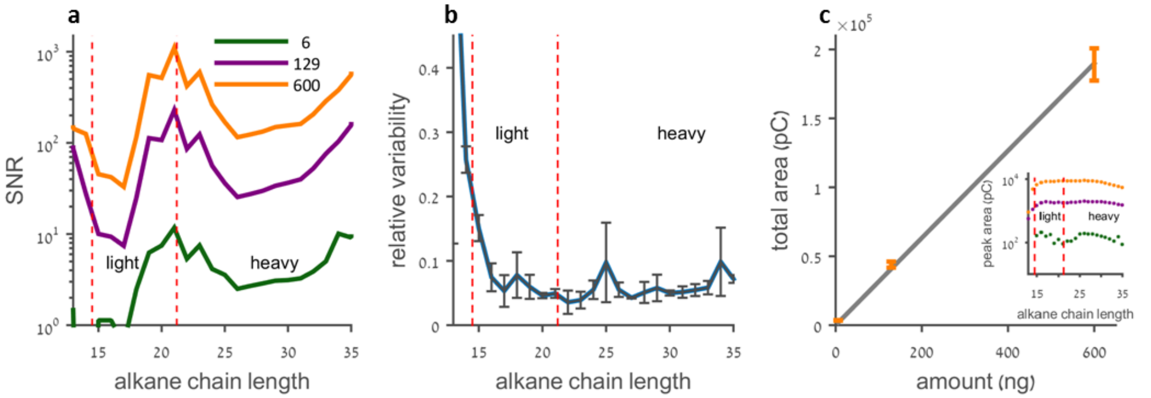

Supplementary figure 3: Method description and verification. (a) SNR measurements for linear hydrocarbon mix of C7:C40 with total amounts of 6, 129 and 600 ng of each compound on a silica plate surface of 20 cm^2 **(b)** Mean coefficient of variation in different chain length. **(c)** GC response to extractions of different total amounts of standards. (c) Inset - GC response measured as peak area to alkanes of varying chain length in three different concentrations (6, 129 and 600 ng of each compound on a silica plate surface of 20 cm^2). For further details see SI section 1.

Task group distribution in asymmetrical nests

The artificial nest structure used for the chemical characterization of nest floors was chosen since it induces a clear association between different chambers and the task groups that occupy them. Indeed, the more internal chambers are occupied by the queen, the brood, and workers with

distended abdomen (the intersegmental membranes become visible giving the abdomen a striped appearance) (72% ± 9%, *N=60* time points in 3 different experiments) that are characteristic of corpulent nurses, whereas the entrance chamber is occupied by non-striped ants (86% ± 6.5%, *N=60* time points in 3 different experiments) associated with lean foragers.

The correlation between body weight and task group affiliation has been previously demonstrated (48). To verify the association between corpulence and stripe pattern the body weight of striped workers 19.3±3.6mg (*N = 10)* was compared to that of plain workers 9±1.3mg (*N = 10).*

Silica plates cleaning procedures

Silica on glass thin layer chromatography (TLC) plates (Analtech) were cut to 6x5 or 3x5 cm2 and placed in one layer in a glass baking pan (Pyrex). The plates were subsequently cleaned using: ethyl acetate, hexane and acetone in the following manner: the plates were covered in solvent and heated to the boiling point of the solvent for 5 minutes. The plates were then transferred to a clean baking pan and the same procedure was repeated with the next solvent.

Supplementary Note 1:

Ants which are associated with the queen's chamber show higher fidelity

Out of the ants that spent over 70% of their time inside the nest 90 where strongly associated with the queen's chamber and 43 with a different chamber. The fidelity of each ant to the floor segment she occupies was evaluated using the manipulation described in Results section "*Nest surfaces affect ant spatial organization*". Each ant was given a score that is a measure of her fidelity to her floor segment (For details see Methods section *"Permutation test").* Ants associated with the queen's room had a mean score of 0.8±0.28 which is significantly different from the scores of the other group 0.4±0.3 (Kolmogorov-Smirnov test, $p<4e-10$). This increased fidelity to the queen's chamber may be attributed to a stronger attraction of the relevant ants to the scents associates with the queen's chamber or perhaps to a tendency of ants to remain for longer periods in denser areas. It cannot be attributed to the attraction of workers to the queen itself since, the queens were not present at the stage in which the ants were reintroduced into the nest.

Supplementary Note 2

Subterranean road signs can be removed by hexane

The raw data for of the experiments described in section "*Hexane soluble compounds as subterranean road signs*" of the main text is shown the tables below.

**Treatment 1:** drying under a nitrogen stream				
	Chamber 1	Chamber 2	Chamber 3	Chamber 4
Experiment 1	19	3	2	0
Experiment 2	26	12	0	4
Experiment 3	22	11	3	2
Experiment 4	17	7	10	7

Supplementary Table 2: ants' distribution in the four nest chambers following treatment 1.

For details see section "*Hexane soluble compounds as subterranean road signs*".

**Treatment 2:** rinsing by sonication in 200ml of hexane followed by drying under a nitrogen stream				
	Chamber 1	Chamber 2	Chamber 3	Chamber 4
Experiment 1	10	1	6	18
Experiment 2	32	2	0	6
Experiment 3	7	7	19	3
Experiment 4	5	11	8	11
Experiment 5	11	16	0	6

Supplementary Table 3: ants' distribution in the four nest chambers following treatment 2.

For details see section "*Hexane soluble compounds as subterranean road signs*".

Supplementary Note 3

Silica plates' floorings induce the same behavior as paper floorings

Silica plates were chosen over paper for the chemical experiments because of their low background noise. In this section we verify that silica plates' floorings accumulate chemical orientation signatures that are recognized by the ants. This is done in order to show continuity between the behavioral experiments presented in section *"Nest surfaces affect ant spatial organization",* which use paper floorings and the chemical experiments presented in "*Classifying chamber function by its chemical signature",* which use silica plates. To do this we housed colonies (N = 4) in symmetric four chambered nests containing silica floorings in which only one chamber was accessible to the ants. After five days the ants were removed from the nests and the silica floorings were transferred to new nest structures in which all chambers were accessible. The ants were then introduced to the new nests and the number of ants in each chamber was counted after 24 hours. In all experiments (N = 6) the chamber that contained the largest fraction of ants was the chamber that was originally accessible. This implies that the silica plates floorings contain an orientation stimulus ($p < 2e\text{-}04$, by the weight of the tail of the corresponding Binomial distribution).

Supplementary Note 4

Refuse area floor extraction

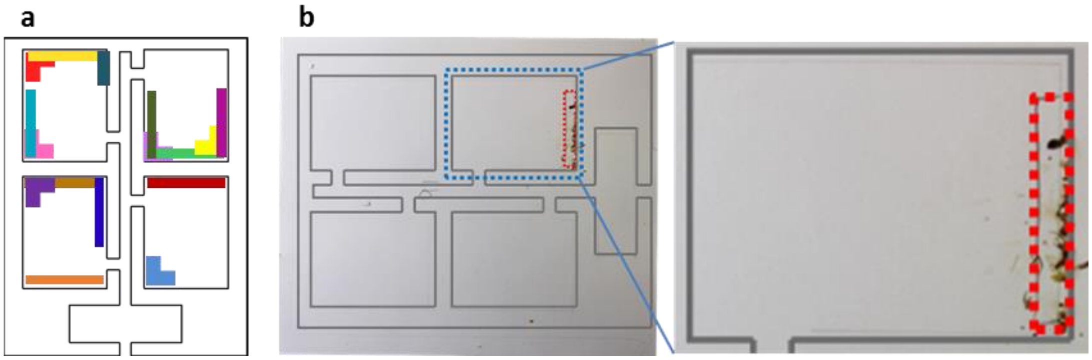

Supplementary figure 4: ants react to refuse pile extraction. a) Refuse pile natural distribution overlay where the refuse pile location of each experiment is uniquely colored **b)** Refuse pile recreated on top of its floor extraction.

Colonies were placed in artificial nests with silica floorings where they created refuse piles which were randomly placed along the walls of the inner chambers (see SI figure 4a). In fact over the 16 experiments we conducted the refuse pile was in the same location only twice. After 5 days, we transferred the colony to a new nest to which a hexane extract taken from the original refuse area was added to a 2-3 cm strip randomly chosen near one of the inner chambers walls (total of 64 cm of nest wall). The ants recreated the new refuse pile on top of the hexane extract in 4 out of 16 repeats (see SI figure 4). Although this is not a high percentage, if one considers all possible locations of the refuse pile, the signal is significantly above random ($p< 0.005$).

Supplementary Note 5

Nest floor chemical composition

A chromatogram of nest floor extraction divided into 'light' and 'heavy' sections used for the chemical classification is presented in SI figure 4. Peaks' identities are listed in the figure caption. Only peaks that were well above the background noise (Kolmogorov Smirnov test, $p<0.05$) were used for the classification. Samples were analyzed on a 7890 Agilent gas chromatograph coupled to a LECO Pegasus time of flight mass spectrometer equipped with a Gerstel cooled injection inlet and a fused silica column (DB5-MS 30 m × 0.25 mm × 0.25 µm, Agilent), as described in section *"GC-MS analysis"* in the Methods. Compound identification was deduced from their mass fragmentation and retention indices as compared to standard compounds when available. It is important to note that some of the prominent compounds of the Dufour's glad of this species, specifically undecane ($H_{11}H_{24}$) and tridecane ($C_{13}H_{28}$) are measured at negligible amounts on nest surfaces due to their higher evaporation rate and low SNR of our method at this mass range.

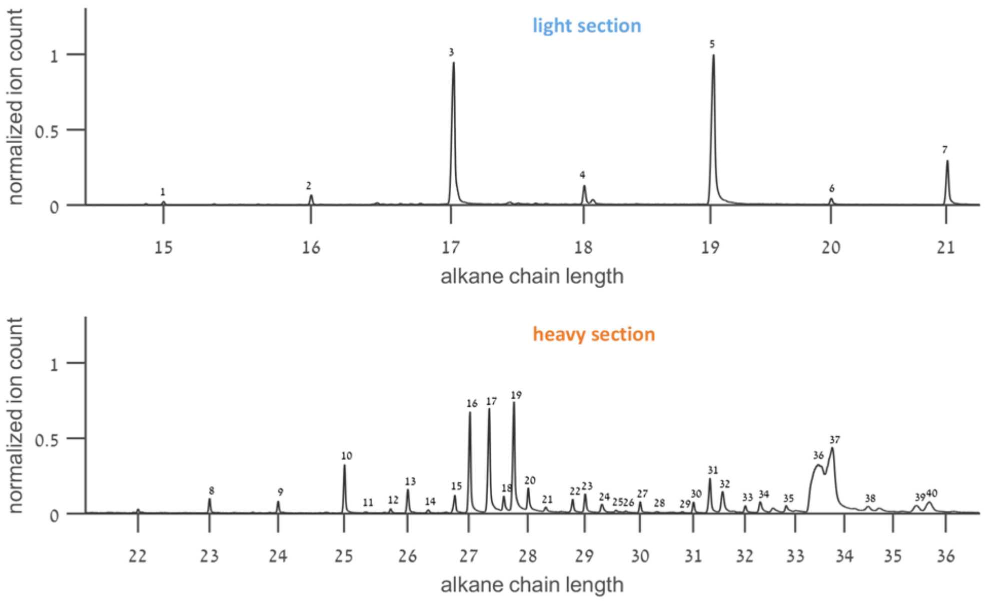

Supplementary figure 5: nest floor hydrocarbon composition chromatogram of nest floor extraction divided into 'light' (top) and 'heavy' (bottom) regions. Marked peaks identity is as follows **(1)** pentadecane **(2)** hexadecane **(3)** heptadecane **(4)** octadecane **(5)** nonadecane **(6)** eicosane **(7)** heneicosane **(8)** tricosane **(9)** tetracosane **(10)** pentacosane **(11)** 11-methyl pentacosane **(12)** 3-methyl pentacosane **(13)** hexacosane **(14)** 12-+14-+16-methyl hexacosane **(15)** heptacosene **(16)** heptacosane **(17)** 11-+13-methyl heptacosane **(18)** 11,15-dimethyl heptacosane **(19)** 3-methyl heptacosane **(20)** octacosane **(21)** 12-+14- methyl octacosane **(22)** nonacosene **(23)** nonacosane **(24)** 11, 13-dimethyl nonacosane **(25)** 11-+15-methyl nonacosane **(26)** 3-mehtyl nonacosane **(27)** triacontane **(28)** 10- + 11- + 12-methyl triacontane **(29)** hentriacontene **(30)** hentriacontane **(31)** 11-methyl hentriacontane **(32)** 11, 15- + 13, 17-dimethyl hentriacontane **(33)** dotriacontane **(34)** 10- + 12-methyl dotriacontane **(35)** tritriacontene **(36)** 11- + 13- + 15-methyl triacontane **(37)** 11,15-dimethyl tritriacontane **(38)** 12-methyl tetratriacontane **(39)** 11- + 13- + 15- + 17- methyl pentatriacontane **(40)** 11, 15-dimethyl pentatriacontane

Supplementary Note 6

Nest surfaces chemical profiles are colony specific

The chemical signature of nest surfaces is colony specific, regardless of the function of the chamber it is taken from. To show that, we performed Principal Component Analysis (PCA) on nest floor samples taken from two different colonies in two experiments. For each colony in each experiment we sampled 7 different chamber floors as described in section *"Classifying chamber function by its chemical signature"*. A total of 28 samples were used for the analysis, 14 of each colony taken during two experiments. Plotting the data in the basis of the first two principal components reveals two clusters that correspond with the two different colonies (see SI figure 11).

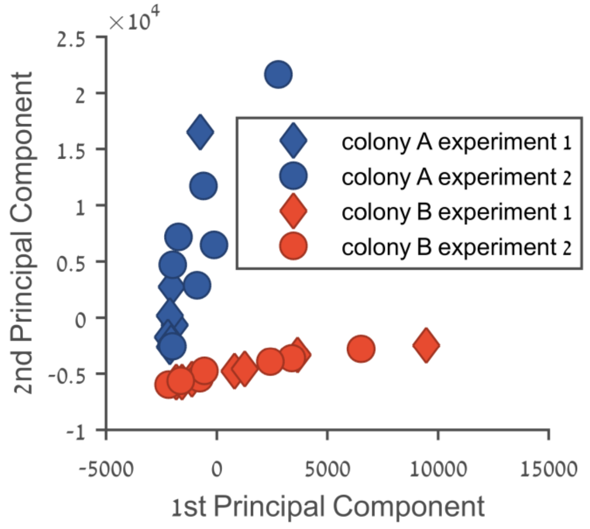

Supplementary figure 6: the chemical signature of the nest surfaces is colony specific. A PCA scatterplot of 28 nest floor samples taken from two colonies over two experiments.

Supplementary Note 7

Refuse area floor has a distinct chemical profile

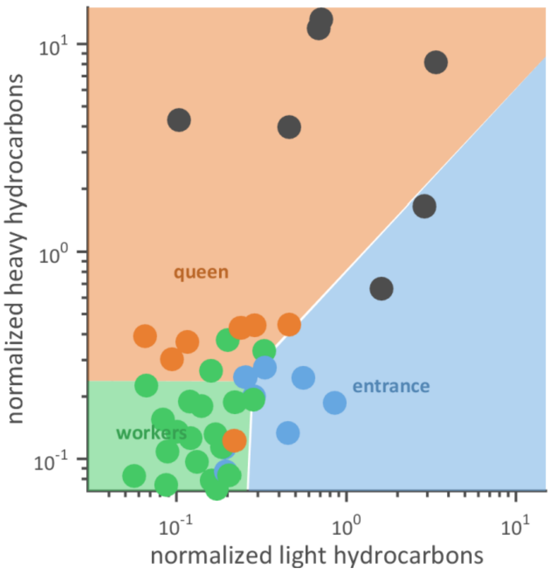

Supplementary figure 7: Refuse area floor chemical profile Values of the 'light' and 'heavy' variables as calculated refuse area samples (gray circles) overlaid on the chambers' classification regions identical to those presented in figure 2c.

Refuse area floor extractions were made in 7 experiments by scraping a floor section of 1 x 1 cm^2 and using the same extraction procedure described in the Methods section. The 'heavy' and 'light' values of each sample were normalized by the sum of the same values in the internal chambers, the same factor used to normalized the internal chambers (sum over all internal chambers, in a specific experiment, adds up to one). The resulting data points were plotted on the chamber classification areas shown in figure 2c. Refuse area data points are clearly separated from other floor data points due to their overall high intensity (see SI figure 6). The high intensity of refuse pile is not the result of high ant occupancy as it covers a small area (~1 x 1 cm^2) which is usually unpopulated. The high hydrocarbon content of this area could the result of accumulation of waste products.

Supplementary Note 8

Queens' CHC profiles do not match the chambers' classification regions

Queen samples had substantially higher intensities in both "light" and "heavy" axes (by an order of magnitude) and therefore were not summed and normalized along with the individual workers samples. Including these samples in the normalization scheme of the individual workers samples wipes out any differences between workers samples. Instead, queen samples were divided by the sums of "light" and "heavy" attributes of the individual workers averaged samples, as explained in Methods section "Direct chemical extraction from ants".

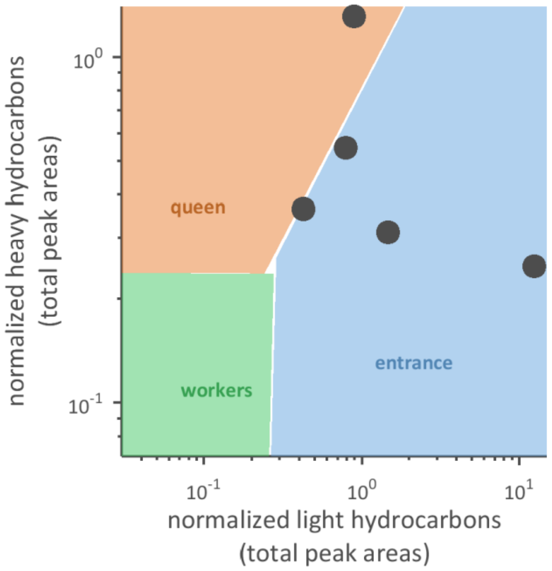

Supplementary figure 8: Queens' CHC profiles do not match the chambers' classification regions Values of the 'light' and 'heavy' variables as calculated for queens (circles) overlaid on the chambers' classification regions identical to those presented in figure 2c.

Supplementary Note 9: Brood cuticular hydrocarbon content is insignificant compared to adult ants

For the preparation of adult and brood extractions two workers and brood of equivalent size in various developmental stages from the same colony were killed by freezing and transferred to vials containing 1 ml hexane immediately. The hexane solutions were transferred to new vials 20 min after the beginning of the extraction. Samples were analyzed on a GC-FID as described in the Methods section. Brood hydrocarbon signal appears to be insignificant compared to adult ants. The raw chromatograms of adult workers and brood extractions are shown in SI figure 8.

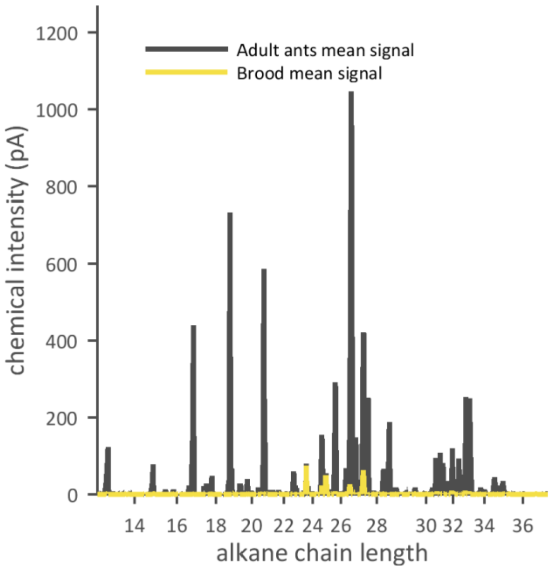

Supplementary figure 9: Brood *vs* adult ants cuticular extracts raw chromatograms of cuticular extracts taken from adult ants (gray) and from brood of various developmental stages (yellow).

Supplementary Note 10

Reliability of SVM results

This section aims to rule out the possibility that the classifier's (overall) performance is driven by specific "strong signals" in the data, *e.g.*, the queen's chamber. Such "masking" may be misleading and to clarify this point we added here the four-class confusion matrix displaying all correct and incorrect classifications (SI table 4). The table displays the number test data points that were assigned to each of the classes ('predicted' classes) together with their true classification ('actual' classes). The diagonal corresponds to true positive classifications. Also shown are the

precision (a.k.a positive predictive value) and recall (a.k.a sensitivity) of each of the four classes over the test set. The four classes are indeed reliably detected by the classifier.

		**predicted**				
		Entrance	Arena	Workers	Queen	**recall**
**actual**	Entrance	2	2	0	0	0.5000
	Arena	0	5	3	0	0.6250
	Workers	0	1	8	3	0.6667
	Queen	0	0	1	3	0.7500
	**precision**	1.0000	0.6250	0.6667	0.5000	**Accuracy** 0.6429

Supplementary Table 4: Confusion matrix over the test data

Had there been less classes, or that one (or more) of the classes would have been incorrect, its test performance would evidently be low. To display this point, the labels of training data from the entrance and arena chambers were randomly shuffled and a four-class SVM was built similarly to the real case.

		**predicted**				
		Entrance	Arena	Workers	Queen	**recall**
**actual**	Entrance	0.29	3.71	0	0	0.0725
	Arena	0.13	2.01	5.86	0	0.2512
	Workers	0	0.01	8.99	3	0.7492
	Queen	0	0	1	3	0.7500
	**precision**	0.69	0.3508	0.5672	0.5000	**Accuracy** 0.5104

Supplementary Table 5: Confusion matrix where “entrance” and “outside” labels were randomly shuffled in the training set

The test data (that retained their correct labels) were classified by the resulting SVM. The values in the four-class confusion matrix that appears below (SI table 5) correspond to averaging 100 such instances of shuffling the arena-entrance labels and then classifying the test data. The precision and recall values of these two classes sharply dropped while the values corresponding to the two other classes remained high. Actually, the "entrance" label almost vanished and most of

its test data points were classified as "arena". These data support the conclusion that four classes are indeed present.

Supplementary Note 11

Predictive compounds strongly correlate with the total amount of heavy compounds

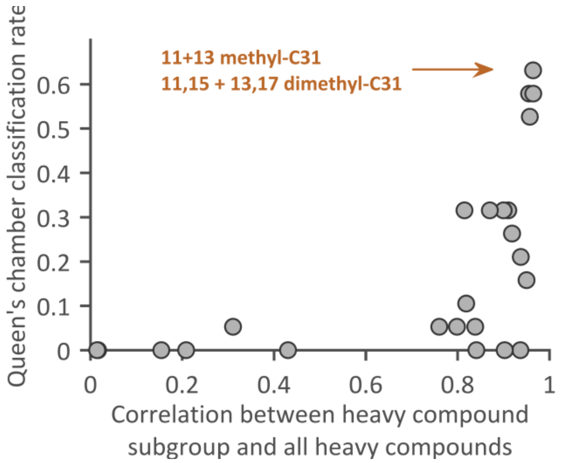

Supplementary figure 10: The predictive power of smaller groups of 'heavy' compounds in classifying the queen's chamber plotted against their correlation with the sum of all 'heavy' compounds (excluding, per each data point, the compounds in the small group in question).

Supplementary Note 12

Substrate background comparison

Comparing hexane extracts of silica plates and filter paper of same surface area we find that filter paper contain significantly higher levels of background noise (see SI figure 12).

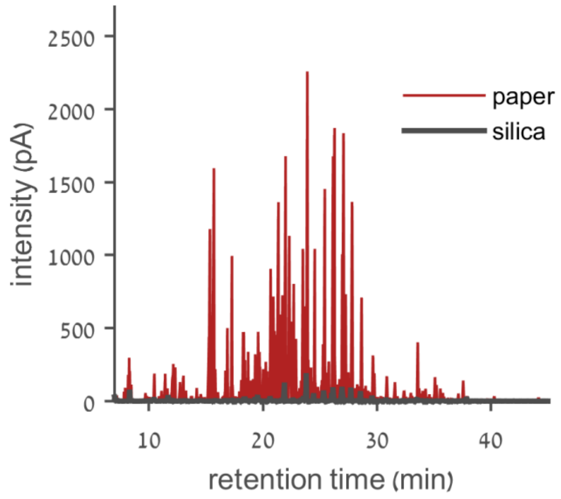

Supplementary figure 11: substrate comparison. Background measurements taken from hexane extractions of paper (red) and silica (grey).

Supplementary Note 13

Binary chamber choice experiment control

In this section we describe control experiments to the experiments described in sections "Manipulating task group positions through floor composition" in the Results and in "Binary chamber choice experiment" in the Methods**.** To test the role of each extract independently and validate the effectiveness of the chosen concentration, we performed experiments in which only one chamber was labeled while the remaining chamber was left unmodified. These control experiments were conducted in the same way described in "Binary chamber choice experiment" in the Methods but leaving one chamber unmodified.

First, we note that for both extracts the labeled chamber draws more attention (higher ant density) than the unmodified chamber in the first 30 minutes right after the introduction of the nest into the arena. We conclude from this that the chosen concentrations of both extracts are effective and recognized by the ants.

Second, the nurses and foragers distribution across the two chambers is affected by the extracts. Nurses' fraction in the chamber labeled with head and thorax extract is higher than their fraction in the empty chamber while in the experiment conducted with Dufour's gland extract the situation is reversed. The results of these experiments are summarized in SI table 6.

To assess the significance of these results we defined 'correctly located' ants to be nursers residing in either un manipulated chambers or chambers labeled with a head + thorax extract and foragers residing in chambers that were either un manipulated or labeled with Dufour's gland extract. All other ants were defined as 'wrongly located'. We then calculated the probability to randomly measuring a given proportion of correctly located ants at a each time point by assuming that individual ants do not distinguish between the labeled and unlabeled chambers, meaning that the probability of an ant to choose a specific chamber is 0.5 regardless of her task group affiliation. To ensure independence of measurements, we performed this calculation on time points that are spaced 1 minute apart. P values were calculated as the weight of the tail of a binomial distribution.

**Label type**	**Head+thorax extract**	**Dufour's gland extract**
Nurses fraction in labeled chamber	0.85	0.56
Nurses fraction in unlabeled chamber	0.26	0.85
Number of time points	21	22
P value	>1e-6	>2e-4

Supplementary Table 6: single extract effect in binary chamber choice. Table shows nurses' fraction across two chambered nests in which one of the chambers is labeled with either head and thorax extract or Dufour's gland extract and the remaining chamber is unmodified (foragers fraction complements the figures presented in the table to one).
